# Tissue Microarray-Based Evaluation of Chromatin Assembly Factor-1 (CAF-1)/p60 as Tumour Prognostic Marker

**DOI:** 10.3390/ijms130911044

**Published:** 2012-09-05

**Authors:** Massimo Mascolo, Gennaro Ilardi, Francesco Merolla, Daniela Russo, Maria Luisa Vecchione, Gaetano de Rosa, Stefania Staibano

**Affiliations:** Department of Biomorphological and Functional Sciences, Pathology Section, University of Naples “Federico II”, Naples 80138, Italy; E-Mails: mmascol@gmail.com (M.M.); gennaro.ilardi@unina.it (G.I.); francesco.merolla@unina.it (F.M.); danielarusso83@yahoo.it (D.R.); marysilvio@alice.it (M.L.V.); gaderosa@unina.it (G.R.)

**Keywords:** Chromatin Assembly Factor-1, tissue microarray, immunohistochemistry, cancer screening

## Abstract

In this study we aimed to confirm the emerging role of Chromatin Assembly Factor 1 (CAF-1 p60) as a new proliferation and prognostic marker for cancer and to test the usefulness of the tissue microarray technique (TMA) for CAF-1 p60 rapid screening in several human malignancies. CAF-1 is a histone chaperone, regulating chromatin dynamics during DNA replication and repair in eukaryotics. TMA is a powerful high-throughput methodology in the study of cancer, allowing simultaneous assessment of different biomarkers within large numbers of tissue specimens. We generated TMA taking 3 mm diameter-core biopsies from oral squamous cell carcinoma, prostate cancer, salivary gland tumours and skin melanoma specimens, which had been previously tested for CAF-1 p60 on routine tissue sections. We also analysed, for the first time, 30 larynx and 30 skin squamous cell carcinomas. CAF-1 p60 resulted over-expressed in both the tissue sections and the TMA specimens, with the highest levels of expression in tumours which were more aggressive and metastasizing. Notably, a high degree of agreement was found between the CAF-1 p60 assessment on TMAs and on routine tissue sections. Our findings confirm the prognostic role of CAF-1 p60 and indicate TMA as a really advantageous method for CAF-1 p60 immunohistochemical screening, allowing savings on both tissue quantity and operator-time.

## 1. Introduction

Cancer, traditionally considered a genetic disease, is now thought to represent the result of a mixture of genetic and epigenetic events, which variously influence the degree of its biological aggressiveness and metastasizing ability [1–8]. The potentially reversible nature of the epigenetic changes has led to hypothesize the use of epigenetic modulators for the establishment of alternative anticancer therapies [9–11]. The epigenetic information includes DNA methylation, RNA-mediated silencing and histone modifications and, although DNA methylation and histone acetylation are among the most frequent epigenetic modifications observed both in normal and neoplastic cells, the disruption of any of these three distinct and mutually reinforcing epigenetic mechanisms might lead to an inappropriate gene expression, resulting in cancer development and other “epigenetic diseases” [9,12]. Epigenetic alterations, especially histone modifications, influence cellular metabolism mainly affecting the chromatin structure. Great attention has been recently focused on the role of chromatin dynamics as a critical determinant in many nuclear events and in pathological conditions such as tumour development and progression [13–15]. Within the cell, the nuclear DNA is tightly packaged in the chromatin structure. The nucleosome is the fundamental unit of chromatin and its core particle is composed of an octamer of the four core histones (H3, H4, H2A, H2B), around which 147 base pairs of DNA are wrapped. DNA packaging follows several orders of wrapping and is fundamental for the maintenance of the genome stability regulating the DNA-based activities as DNA replication, transcription and repair. Histone chaperons play a critical role in maintaining and regulating chromatin structure by driving histones deposition. CAF-1 (Chromatin Assembly Factor-1) is a heterotrimeric histone chaperone formed by p48, p60 and p150 proteins that plays a pivotal role in the regulation of chromatin assembly coupled to DNA replication and repair [16]. During the S phase of the eukaryotic cell cycle, the newly replicated DNA is rapidly assembled into chromatin. CAF-1 mediates the deposition of newly synthesised histones H3.1 and H4 onto nascent DNA [17] and their assembly into nucleosomes, by association with PCNA [18]. In the same way, CAF-1 complex mediates the assembly of nucleosomes following DNA damage repair. Several reports have recently shown that a deregulated expression of CAF-1 p60 is linked to the neoplastic progression of most of the human solid malignancies [17,19–22]. In particular, the CAF-1 p60 subunit has been found overexpressed in breast, oral, prostate, and salivary gland carcinomas, as well as in skin melanoma [23–28]. Interestingly, CAF-1 p60 expression levels are significantly correlated with the biological aggressiveness of tumours, metastasizing behaviour and worse prognosis [23–28]. This suggested CAF-1 p60 could have a promising role as a new sensible “transversal” prognostic marker for human tumours, apparently unrelated to their histogenesis. To date the *on-site* expression of CAF-1 p60 in human tumours has been mostly performed on routine sections of paraffinized tissues; in addition, fine needle aspirates have also been used in order to evaluate CAF-1 p60 expression at least in breast [27] and salivary gland tumours [23]. In the present study, we evaluated the immunohistochemical expression of CAF-1 p60 on tissue microarray (TMA) sections generated by taking core biopsies from the same tumour series previously evaluated for this protein. The aim of our study is to assess the degree of agreement in the extent and intensity of CAF-1 p60 immunoreactivity between the values observed in TMAs and routine sections. As is well known, TMA constitutes a powerful high-throughput methodology which has been increasingly used for validation of new cancer biomarkers, and hopefully it could represent a valuable tool for the rapid screening for CAF-1 p60 expression in malignant tumours, in the context of their prognostic evaluation.

## 2. Results

### 2.1. CAF-1 p60 in Normal Tissue

CAF-1 p60 immunostaining showed a focal, scattered, nuclear positivity in TMAs as in routine sections of normal tissue specimens. CAF-1 p60 positive cells were found almost always localized in the regenerative compartment: from 0 to <10% of cells from the basal layer of epidermis and oral mucosal epithelium keratinocytes, of melanocytes at the dermal-epidermal junction, or of secretory cells of prostate and salivary glands showing, in fact, CAF-1 p60 positivity at immunostaining.

### 2.2. CAF-1 p60 in Tumours

**Figure 1:**
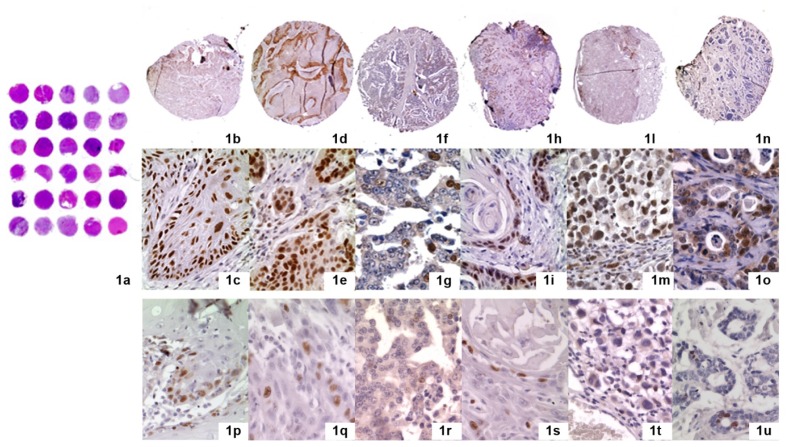
Immunohistochemical staining for Chromatin Assembly Factor 1 (CAF-1 p60) (LSAB technique) (**a**) the design of tissue microarray technique (TMAs) (haematoxylin and eosin staining). Each slide contains 5 × 6 cores (30 cores) sampled from neoplastic tissues; (**b**,**c**) Oral Squamous Cell Carcinoma (OSCC), b: 25×, c: 250×; (**d**,**e**) Laryngeal Squamous Cell Carcinoma (LSCC), d: 25×, e: 250×; (**f**,**g**) Prostate Carcinoma (PC), f: 25×, g: 250×; (**h**,**i**) Skin Squamous Cell Carcinoma (SSCC), h: 25×, i: 250×; (**l**,**m**) Skin Melanoma (SM), l: 25×, m: 250×; (**n**,**o**) Salivary gland tumours (SGT), n: 25×; o: 250×. Ki67 immunohistochemical staining for Ki67 (LSAB technique) is shown in **p** (OSCC), **q** (LSCC), **r** (PC), **s** (SSCC), **t** (SM) and **u** (SGT).

**Figure 2:**
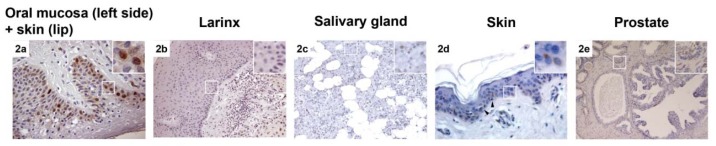
Immunohistochemical staining for CAF-1 p60 (LSAB technique) on normal tissues (**a**) Lower lip, muco-cutaneous junction (left: oral mucosa; right: skin); (**b**) Larynx: normal-to hyperplastic, with underlying infiltrating squamous cell carcinoma; (**c**) Salivary gland; (**d**) Skin (arrow heads indicate normal melanocytes negative to p60 immunostaining); (**e**) Prostate glands, normal to hyperplastic. The insert shows magnification of the marked area (white square).

**Figure 3:**
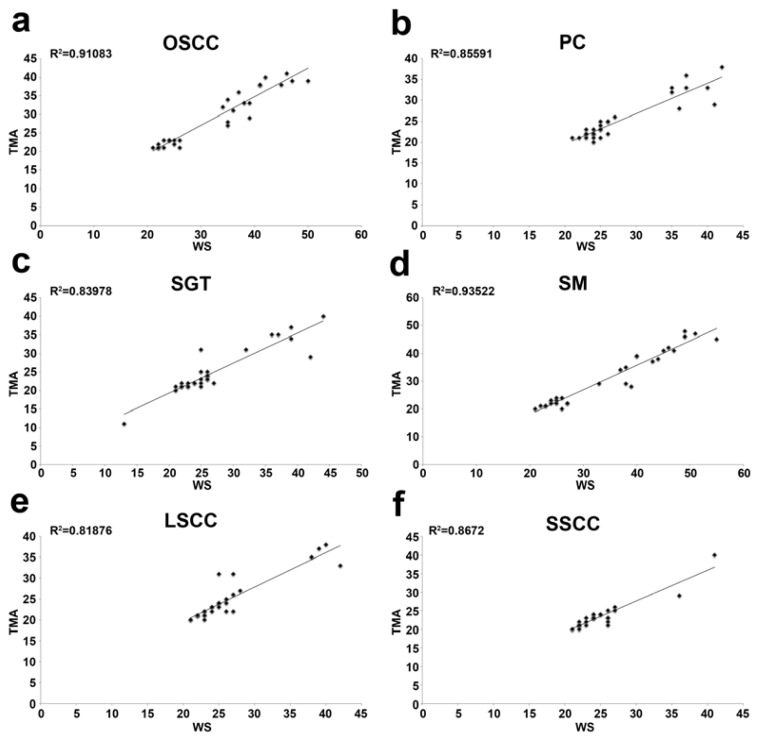
Comparison of the percentage of labelled cells on TMA sections and whole-tissue sections (WS) for the CAF-1 p60 antibody. Scatter plots show a tight grouping of points when the percentage of labelled cells on TMA sections is plotted against the WS for the CAF-1 p60 antibody. The added lines are the lines of best fit, *R*^2^ values of linear regressions are shown. (**a**) Oral squamous cell carcinoma (OSCC); (**b**) prostate cancer (PC); (**c**) salivary gland tumour (SGT); (**d**) skin melanoma (SM); (**e**) laryngeal squamous cell carcinoma (LSCC); (**f**) skin squamous cell carcinoma (SSCC).

All the evaluated malignant tumours showed CAF-1 p60 overexpression (Figure 1, Tables 1–7). Representative images of CAF-1 p60 expression in normal tissues are shown in Figure 2. In detail, a moderate expression (++) of CAF-1 p60 was found in 13 Oral Squamous Cells Carcinoma (OSCC) (3 G1, 8 G2, 2 G3), 22 Prostate Cancer (PC) (1 with Gleason score <7, 15 with a Gleason score equal to 7, of which 5 with a primary pattern of 4 and 10 with a primary pattern of 3, and 6 with Gleason score >7), 14 Skin Melanoma (SM) (4 with Breslow vertical phase thickness <1.00 mm, 3 comprised between 1.01 and 2.00, 4 comprised between 2.01 and 4.00 and 3 > 4.00 mm), 22 Salivary Gland Tumour (SGT), of which 1 polymorphous low-grade carcinoma (PLGC), 3 acinic cell carcinomas (AC), 3 adenoid cystic carcinomas (ACC), 11 muco-epidermoid carcinomas (4 low grade, 3 intermediate-grade and 4 high-grade tumours), and 4 cases of carcinoma ex-PA (CXPA) 26 Laryngeal Squamous Cell Carcinoma (LSCC) (6 G1, 6 G2, 14 G3) and 28 Skin Squamous Cell Carcinoma (SSCC) (8 G1, 10 G2, 10 G3); as a high level of expression (+++) was observed in the remaining 17 OSCC (7 G1, 7 G2 and 3 G3), 8 PC (2 with Gleason score <7, 3 with a Gleason score equal to 7, with a primary pattern of 4, and 3 with Gleason score >7), 16 SM (2 with Breslow vertical phase thickness <1.00 mm, 5 comprised between 1.01 and 2.00, 6 comprised between 2.01 and 4.00 and 3 > 4.00 mm), 7 cases of malignant SGT, of which 1 adenoid cystic carcinomas (ACC), 5 muco-epidermoid carcinomas (1 low grade, 3 intermediate-grade and 1 high-grade tumours) and 1 case of CXPA, 4 LSCC (1 G1, 1 G2, 2 G3) and 2 SSCC (1 G2 and 1 G3). The values found on the whole sections of OSCC, PC, SGTs and SM were in agreement with those already reported in the literature [23–26]. The evaluation of the immunohistochemical expression of CAF-1 p60 on TMA sections of the same tumour series gave rise to quite similar results, with an excellent level of agreement for both the intra- and inter-observer evaluation of the expression of CAF-1 p60 on the whole sections and TMAs (K-coefficient: 0.8018 for OSCC; 0.8148 for PC; 0.8018 for SM; 0.8529 for SGT; 0.8696 for LSCC, 0.8076 for SSCC) (Table 8, Figure 3). According to univariate statistical analysis, both on routine whole sections and on TMA, no statistical correlation was found between CAF-1 p60 expression, age and sex of patients (data not shown).

**Figure 4:**
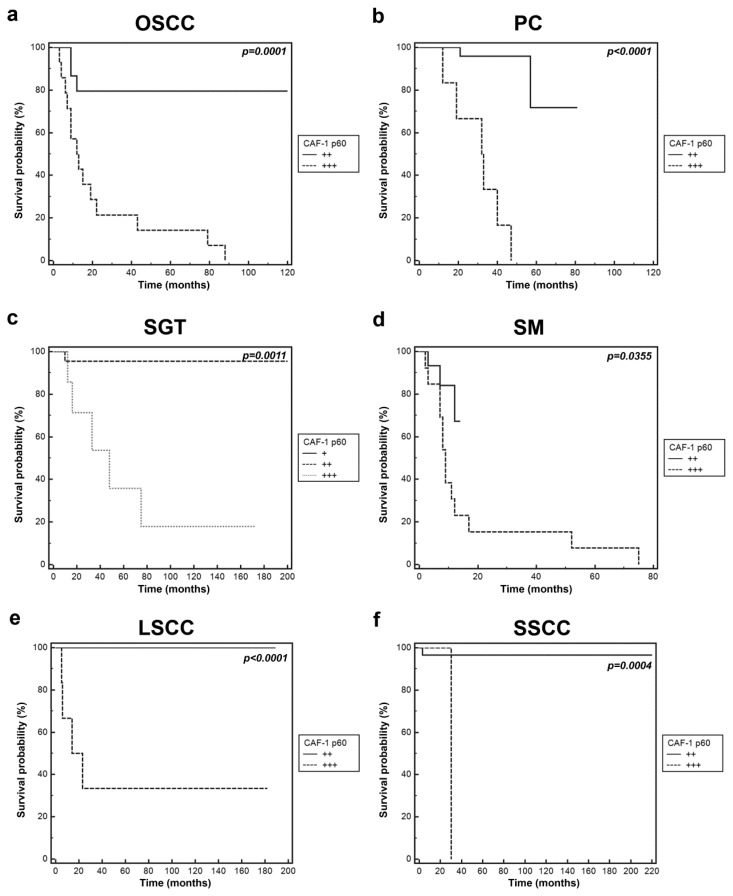
Kaplan-Meier plots showing disease free survival for OSCC, PC, SGT, SM, LSCC AND SSCC patients grouped by the level of expression of CAF-1 p60. Tumor samples were stratified in three categories (+, ++, and +++) based on intensity of CAF-1 p60 immunostaining. The comparison between survival curves and *p* value was determined by a two-sided log-rank test. (**a**) Oral squamous cell carcinoma (OSCC); (**b**) prostate cancer (PC); (**c**) salivary gland tumour (SGT); (**d**) skin melanoma (SM); (**e**) laryngeal squamous cell carcinoma (LSCC); (**f**) skin squamous cell carcinoma (SSCC).

Irrespective of the tumour histogenesis, CAF-1 p60 showed high levels of expression in all evaluated tumours (OSCC, PC, SM, SGTs and LSCC and SSCC) with the highest values in cases showing adverse events during the follow up (relapse, lymph node and/or distant metastasis, death from disease). Correlation between CAF-1 p60 expression and adverse events at follow-up proved to be statistically significant, as shown by Kaplan-Meier curves in Figure 4.

## 3. Discussion

The high concordance in the evaluation of CAF-1 p60 expression between the TMAs and the whole section specimens, in agreement with data reported from similar comparative studies in literature [29–33], supports the idea that the TMA technique is a very useful tool for novel diagnostic and/or prognostic marker screening in tumours. The TMA method, used in the majority of pathology laboratories, is a standardized technology based on the original technique proposed by Wan *et al.* and further modified by Kononen in 1998 [34,35]. Recently, many studies, comparing the data obtained from conventional and TMA sections, validated the use of TMAs as a useful tool for diagnostic and/or prognostic biomarkers screening in several tumours, such as oral and oesophageal squamous cell carcinomas [36,37], ovarian cancers [38], breast carcinomas [39], and peripheral nerve malignant sheath tumours [40]. This report constitutes the first study in which the TMA method was used to evaluate the immunohistochemical expression of CAF-1 p60 in different human malignancies and correlated with adverse clinical outcome. Our data obtained on sections of TMAs confirmed that CAF-1 p60 protein is increased in all the evaluated tumours, from those that have already been reported in the literature, such as MM, PC, OSCC and OSGT, to the newly investigated LSCC and SSCC. Recently, Polo and coll. published a study showing the overexpression of CAF/p60 in a series of different human malignancies [28]. In the present study, we have shown that in our selected series of patients, the higher expression of CAF-1 p60 was significantly associated to a more aggressive biological behaviour of tumours, confirming the idea that the increase of CAF-1 p60 expression is strictly correlated with the cancer progression and the metastasizing ability of transformed cells, irrespective of their histological type and differentiation. Notably, our data strongly suggest that the use of TMAs is a reliable technique for the immunohistochemical assessment of CAF-1 p60 expression, and it may be proposed as an efficient, time- and cost-effective, evaluation of this new promising biomarker of the aggressiveness of malignant tumours [41–43]. Moreover, as multiple recent reports have indicated the immunohistochemical evaluation of CAF-1 p60 expression may add important information to the prognostic prevision of most human solid malignancies; the finding that this evaluation can be performed on TMAs with results quite similar to those derived from the immunostaining of routine whole tissue sections is extremely exciting, in terms of use of both pathologists’ time and resources. In addition, the TMA assembly procedure used for this study is easy to perform and cheaper than that obtained with the use of the expensive automated tissue array devices. For this reason, the manual TMA technique can be used even in small pathology laboratories with a limited budget, representing the ideal pre-requisite for a screening technology. Using 3 mm diameter tissue cores we can strongly reduce the bias due to the reduced amount of tissue, especially when compared to the TMA made of small cores obtained with automatized devices from hundreds of individual specimens. In addition, the time- and cost-efficient manual TMAs based on large 3 mm cores, allow a better evaluation of both the histological characteristics, as well as the extent and pattern of the immunostaining signal distribution. A single 3 mm-diameter tissue core provides, in fact, more than twice the total tumour area covered by the three small cores obtained with the automatized systems. TMA is a time- and cost-efficient method of evaluating the immunohistochemical expression of several proteins in a large number of tumours. The TMA technique leads, in fact, to great economy in time, reagents and tissue specimens and facilitates the rapid standardization and introduction of new antibodies into routine diagnostic immunohistochemistry, allowing pathologists to quickly evaluate hundreds of cores of selected tissue samples [44,45]. Moreover, the TMA technique provides a great opportunity to easily analyse, store and share IHC data on a large number of samples over a long period of time when combined, with the digital management of slides, offering a cost-efficient alternative to routine diagnostics.

## 4. Experimental Section

In the present study, we selected a total of 180 human tumours diagnosed at the Department of Biomorphological and Functional Sciences, Pathology Section, University “Federico II” of Naples, Italy, from 1 January 2000 to 31 December 2009. The selected cases encompassed 30 oral squamous cell carcinomas (OSCC), 30 prostate carcinomas (PC), 30 salivary gland tumours (SGT) and 30 skin melanomas (SM), all belonging to the same selected series of patients who had previously been screened for CAF-1 p60 by immunohistochemistry [23–26] and 30 laryngeal squamous cell carcinomas (LSCC) and 30 skin squamous cell carcinomas (SSCC), that had never been evaluated for CAF-1 p60 expression. The clinic-pathological features of all patients are summarized in Table 1. For each patient, the tumour stage class was determined according to the American Joint Committee, AJCC [46]. The study was performed according to the Declaration of Helsinki and in agreement with Italian law that, due to the topics of this research, does not provide a specific Ethical Committee assent.

### 4.1. TMAs Construction and Immunohistochemistry

Two pathologists (SS and MM) reviewed the whole routine haematoxylin-eosin (H & E) sections to confirm the original diagnosis and to mark the most representative tumour areas useful for the TMA construction. From one paraffin block of each tumour, a 4-μm thick section was cut and mounted on a slide pre-treated for immunohistochemical evaluation of CAF-1 p60 expression. Then, for all selected cases, the tumour area for TMA construction was identified on the same paraffin donor blocks under the guidance of the corresponding previously marked H & E section and punched by a manual tissue-array instrument (Tissue-Tek Quick-Ray, Sakura Finetek, Torrance, CA, USA). The tissue cores (3 mm in diameter) were carefully transferred to the recipient paraffin blocks with 30 holes each. The filled recipient blocks were then placed on a metal base mould. The paraffin-embedding was then carried-out, by heating the blocks at 42 °C for 10 min, and flattening their surface by pressing a clean glass slide on them. As a result, a TMA was built for each cancer histotype (OSCC, LSCC, PC, SSCC, SGT and MM). In addition, cores of non-neoplastic oral and laryngeal mucosa, salivary glands, prostate tissue, and skin were included in a further tissue array block to pursue normal controls. After chilling the tissue arrays to −10 °C for 30 min, according to the technique described two 4-μm sections were cut from each TMA using an ordinary microtome [44]. The first section was stained with H&E to confirm the presence of tumour and the integrity of tissues. The second section was mounted on a super frost slide (Microm, Walldorf, Germany) for the immunohistochemical evaluation of CAF-1 p60 expression. The slides were de-waxed by heating at 55 °C for 30 min followed by 3 washes, 5 min each, with xylene, and rehydrated by 5-min washes with 100%, 95%, and 80% ethanol up to pure distilled water. The Antigen retrieval was obtained by heating the sections at 95 °C for 30 min in 10 mM sodium citrate (pH 6.0). Endogenous peroxidase activity was blocked by the incubation in 3% hydrogen peroxide for 30 min. The background reactivity was removed using the universal blocking serum (Dako Diagnostics, Glostrup, Denmark) for 30 min at room temperature. At this point, the slides were incubated overnight at 4 °C with anti-CAF-1 p60 antibody (SS53-ab8133, Abcam, Cambridge, MA, USA, diluted 1:300) [23–26]. The incubation for 30 min with a biotin-labeled secondary antibody was then performed. The streptavidin-peroxidase (DAKO Diagnostics) was applied and developed using 3,3′-diaminobenzidine substrate (Vector Laboratories, Burlingame, CA, USA). After slight counterstaining with haematoxylin, the slides were dehydrated and mounted with cover slips for microscopic examination. Breast carcinoma sections were used as positive control. Negative controls were included incubating the sections with pre-immune serum instead of the antibody. The cells with a definite brown nuclear staining were judged positive for CAF-1 p60 antibody. The evaluation of nuclear CAF-1 p60 staining was blindly and independently examined in all TMA cores and in whole sections of the selected donor blocks. CAF-1 p60 expression was evaluated as the percentage of positive tumor cells among the total neoplastic cells present in at least 10 high power fields and graded semi-quantitatively according to an arbitrary scale, as follows: 0 (<10% of positive cells); + (10% to <20%); ++ (20% to <30%); +++ (≥30% of positive cells) [23–26]. In case of discrepancy between the two pathologists, the immunostained slides were reviewed in a double viewing microscope to settle the discrepancy. Cores were considered lost if <10% of cells contained tumour (“sampling error”) or when <10% of tissue was present (“absent core”) [47]. Immunohistochemistry was performed with the same end-quality both on whole sections and on TMA, without losing tissue cores during all the steps of the procedure.

### 4.2. Statistical Analysis

Statistical analysis was performed with SPSS package for Windows (release 17.0). The χ^2^ test was used to compare the quantitative differences of CAF-1 p60 staining in various stages of different tumours progression. The Kaplan-Meier curves were used to evaluate the disease free survival of patients grouped by CAF-1 p60 expression. A two-sided Log-rank test was used to compare Kaplan-Meier curves and to assess statistical significance. The *p-*value was considered significant if <0.05. To determine the chance-corrected agreement between the immunohistochemical staining scores of TMA core and the whole sections, the Cohen’s weighted kappa statistic was calculated. Chance-corrected agreement was considered poor if *K* < 0.00, slight if *K* was between 0 and 0.20, fair if *K* was between 0.21 and 0.40, substantial if *K* was between 0.61 and 0.80, and almost perfect if *K* was >0.80. The overall agreement was defined as the percentage of correct concordance between the TMA and the donor blocks from the total number of cases [48].

## 5. Conclusions

There is an urgent need for new, reliable prognostic markers to identify human cancers with an aggressive behaviour, and CAF-1 p60 proved to be a very promising candidate, as we have previously shown. So far, CAF-1 p60 expression assessment has been carried out mainly on whole tissue sections. The 3 mm cores TMA is a powerful, cost-effective, high-throughput methodology in the study of cancer, allowing simultaneous, highly accurate, assessment of different biomarkers within large numbers of tissue specimens. In the present study a high concordance was found between CAF-1 p60 assessment on TMAs and on routine tissue sections.

We strongly believe that the manual construction procedure of TMA will facilitate its set-up, easily allowing an enlargement of the amount of data concerning the role of CAF-1 p60 expression in human tumours, favouring the diffusion of this biomarker into the clinical setting.

## Figures and Tables

**Table 1:** Clinical and pathological features of all examined patients.

Histotype	*N*°	*m*	*f*	Age Range (average)	F-up Range (average)	Grade	Outcome
**OSCC**	30	18	12	48–95 years (66.7)	4–120 months (49.23)	10 G1 (33%)	3 NED, 2 M, 1 D, 1 M,D, 3 R,M,D
						15 G2 (50%)	8 NED, 3 M,D, 2 R,D, 2 R,M,D
						5 G3 (17%)	2 NED, 1 M,D, 1 R,D, 1 R,M,D

**PC**	30	30	0	55–80 years (66.7)	30–64 months (40)	3 GS ≤ 6 (10%)	1 NED, 1 M, 1 M,D
						18 GS 7 (60%)	15 NED, 2 M, 1 M,D
						9 GS > 8 (30%)	6 NED, 2 M, 1 M,D

**SM**	30	15	15	21–81 years (46.2)	1–22 years (8.67)	6 BT < 1 (20%)	4 NED, 1 R, 1 N
						8BT 1.01–2 (27%)	3 NED, 3 N, 1 R,N, 1 N,M,D
						10 BT 2.01–4 (33%)	4 NED, 1 M, 2 N, 1 N,M, 2 N,M,D
						6 BT > (20%)	3 NED, 2 N, 1 R,M,D

**SGT**	30	12	18	18–80 years (48.77)	12–200 months (67)	1 PLGC (3%)	1 NED
						4 ACC (13%)	3 NED, 1 M,R
						16 MEC (54%)	12 NED, 3 R, 1 M
						3 AC (10%)	3 NED
						5 CXPA (17%)	4 NED; 1 R,N
						1 PA (3%)	1 NED

**LSCC**	30	30	0	38–76 years (63.3)	38–189 months (130.33)	7 G1 (23%)	6 NED, 1 M
						7 G2 (23%)	6 NED, 1 M
						16 G3 (54%)	14 NED, 2 M

**SSCC**	30	17	13	32–95 years (68.04)	12–223 months (138.83)	8 G1 (26%)	8 NED
						11 G2 (37%)	10 NED, 1 R,N
						11 G3 (37%)	10 NED, 1 R

GS: Gleason score; BT: Breslow thickness; NED: not evidence of disease; R: relapse; M: distant metastasis; D: death for disease; N: nodes metastasis; OSCC: Oral Squamous Cell Carcinoma; PC: Prostate Carcinoma; SM: Skin Melanoma; SGT: Salivary Gland Tumour; LSCC: Laryngeal Squamous Cell Carcinoma; SSCC: Skin Squamous Cell Carcinoma.

**Table 2:** Clinical and pathological features of the study population of OSCC, ordered by grading. Correlation with CAF-1 p60 expression.

Sex	Age	Histotype	Grading	TNM Stage	WS CAF-1 p60	WS CAF-1 p60 (%)	TMA CAF-1 p60	TMA CAF-1 p60 (%)	F-up
m	95	SCC	1	I	++	25	++	22	17 NED
m	64	SCC	1	I	++	22	++	21	21 NED
f	81	SCC	1	I	++	23	++	21	4 NED
m	49	SCC	1	I	+++	34	+++	32	43 D
m	66	SCC	1	III	+++	39	+++	33	120 M
m	49	SCC	1	III	+++	41	+++	38	79 M
m	86	SCC	1	III	+++	42	+++	40	22 M,D
m	75	SCC	1	IV	+++	45	+++	38	11 R,M,D
m	56	SCC	1	IV	+++	47	+++	39	77 R,M,D
f	68	SCC	1	III	+++	46	+++	41	88 R,M,D
m	66	SCC	2	II	++	23	++	23	120 NED
m	50	SCC	2	I	++	22	++	21	109 NED
m	78	SCC	2	III	++	25	++	23	11 NED
m	48	SCC	2	I	++	21	++	21	14 NED
m	48	SCC	2	I	++	24	++	23	85 NED
f	74	SCC	2	I	++	26	++	21	19 NED
f	67	SCC	2	I	++	22	++	22	97 NED
f	59	SCC	2	II	++	24	++	23	98 NED
m	69	SCC	2	III	+++	35	+++	34	12 R,D
m	58	SCC	2	IV	+++	36	+++	31	8 R,D
f	77	SCC	2	I	+++	38	+++	33	114 M,D
f	75	SCC	2	IV	+++	37	+++	36	27 M,D
f	73	SCC	2	III	+++	41	+++	38	10 M,D
m	65	SCC	2	III	+++	35	++	27	20 R,M,D
m	60	SCC	2	IV	+++	50	+++	39	24 R,M,D
f	60	SCC	3	I	++	26	++	23	106 NED
m	66	SCC	3	I	++	22	++	21	97 NED
f	80	SCC	3	II	+++	35	++	28	23 R,D
m	63	SCC	3	III	+++	39	++	29	17 M,D
m	65	SCC	3	II	+++	41	+++	38	15 R,M,D

NED: not evidence of disease; R: relapse; M: distant metastasis; D: death for disease.

**Table 3:** Clinical and pathological features of the study population of PC, ordered by Gleason Score. Correlation with CAF-1 p60 expression.

Sex	Age	Histotype	Gleason	TNM Stage	WS CAF-1 p60	WS CAF-1 p60 (%)	TMA CAF-1 p60	TMA CAF-1 p60 (%)	F-up
m	70	AC	5 (3 + 2)	pT2bN0	+++	36	++	28	57 M
m	51	AC	5 (3 + 2)	pT2bN0	+++	40	+++	33	50 M,D
m	72	AC	6 (3 + 3)	pT2bN0	++	23	++	22	81 NED
m	68	AC	7 (3 + 4)	pT3aN0	++	25	++	25	49 NED
m	67	AC	7 (4 + 3)	pT3aNx	++	27	++	26	33 NED
m	58	AC	7 (3 + 4)	pT2bN0	++	24	++	21	32 NED
m	45	AC	7 (3 + 4)	pT2bN0	++	23	++	23	30 NED
m	53	AC	7 (3 + 4)	pT2bN0	++	24	++	22	75 NED
m	65	AC	7 (4 + 3)	pT4N1	+++	37	+++	36	49 M
m	63	AC	7 (4 + 3)	pT3aN0	++	24	++	23	32 NED
m	68	AC	7 (4 + 3)	pT3bN0	++	26	++	25	33 NED
m	73	AC	7 (3 + 4)	pT3aN1	++	23	++	22	56 NED
m	71	AC	7 (4 + 3)	pT3bN1	++	26	++	25	30 NED
m	80	AC	7 (4 + 3)	pT3bN1	++	24	++	20	40 NED
m	57	AC	7 (3 + 4)	pT2bN0	++	21	++	21	33 NED
m	70	AC	7 (3 + 4)	pT2aN0	++	25	++	23	32 NED
m	56	AC	7 (3 + 4)	pT2bN0	++	23	++	22	33 NED
m	61	AC	7 (3 + 4)	pT2aN0	++	25	++	24	32 NED
m	65	AC	7 (4 + 3)	pT3bN1	+++	41	++	29	43 M,D
m	60	AC	7 (3 + 4)	pT2aN0	++	24	++	22	33 NED
m	62	AC	7 (4 + 3)	pT3aNo	+++	35	+++	33	64 M
m	64	AC	8 (4 + 4)	pT2bN0	++	23	++	21	59 NED
m	66	AC	8 (4 + 4)	pT3bN1	++	26	++	22	33 NED
m	71	AC	8 (4 + 4)	pT3bN1	+++	42	+++	38	57 M,D
m	60	AC	8 (4 + 4)	PT3aN0	++	25	++	21	33 NED
m	70	AC	8 (4 + 4)	pT3bN0	++	22	++	21	32 NED
m	74	AC	8 (4 + 4)	pT3aN0	++	25	++	23	32 NED
m	64	AC	8 (4 + 4)	pT3bN0	+++	37	+++	33	48 M
m	57	AC	8 (4 + 4)	pT3aN0	++	24	++	22	30 NED
m	73	AC	8 (4 + 4)	pT2bN1	+++	35	+++	32	34 M

NED: not evidence of disease; R: relapse; M: distant metastasis; D: death for disease.

**Table 4:** Clinical and pathological features of the study population of SGT, ordered by histology subtype. Correlation with CAF-1 p60 expression.

Sex	Age	Histotype	Grading	TNM Stage	WS CAF-1 p60	WS CAF-1 p60 (%)	TMA CAF-1 p60	TMA CAF-1 p60 (%)	F-up
m	49	PLGC	-	pT1N0M0	++	24	++	22	26 NED
f	33	AC	-	pT3N0M0	++	25	++	21	67 NED
m	45	AC	-	pT2NxM0	++	26	++	24	70 NED
f	78	AC	-	pT3NxM0	++	21	++	20	44 NED
f	39	ACC	-	pT2N0M0	++	25	+++	31	29 NED
f	41	ACC	-	pT3N0M0	++	26	++	24	54 NED
m	48	ACC	-	pT4aNxM0	+++	39	+++	37	71 M,R
f	63	ACC	-	pT2NxM0	++	25	++	22	44 NED
f	20	LG-MEC	low	pT1N0M0	++	22	++	21	49 NED
f	57	LG-MEC	low	pT3N0M0	+++	32	+++	31	173 NED
f	18	LG-MEC	low	pT2N0M0	++	26	++	23	92 NED
f	40	LG-MEC	low	pT4aN0M0	++	27	++	22	69 NED
f	62	LG-MEC	low	pT3NxM0	++	23	++	22	103 NED
m	39	IG-MEC	intermediate	pT2N0M0	+++	37	+++	35	140 R
f	51	IG-MEC	intermediate	pT2N2bM0	+++	39	+++	34	132 M
m	57	IG-MEC	intermediate	pT1NxM0	+++	44	+++	40	94 R
m	72	IG-MEC	intermediate	pT1NxM0	++	25	++	23	31 NED
f	74	IG-MEC	intermediate	pT1NxM0	++	21	++	21	95 NED
f	80	IG-MEC	intermediate	pT3NxM0	++	26	++	24	97 NED
m	56	HG-MEC	high	pT2N0M0	+++	36	+++	35	131 R
f	32	HG-MEC	high	pT4NxM0	++	25	++	25	200 NED
f	33	HG-MEC	high	pT1NxM0	++	22	++	21	23 NED
m	51	HG-MEC	high	pT2N0M0	++	24	++	22	72 NED
m	60	HG-MEC	high	pT3N0M0	++	26	++	23	34 NED
f	56	CXPA	-	pT2NxM0	++	23	++	21	23 NED
f	24	CXPA	-	pT2N0M0	++	26	++	25	97 NED
m	42	CXPA	-	pT1N0M0	+++	42	++	29	55 R,N
m	43	CXPA	-	pT3N0M0	++	23	++	21	63 NED
m	72	CXPA	-	pT2NxM0	++	22	++	22	166 NED
f	28	PA	-	-	+	13	+	11	12 NED

NED: not evidence of disease; R: relapse; M: distant metastasis; D: death for disease.

**Table 5:** Clinical and pathological features of the study population of SM, ordered by Breslow thickness. Correlation with CAF-1 p60 expression.

Sex	Age	Histotype	Breslow	TNM Stage	WS CAF-1 p60	WS CAF-1 p60 (%)	TMA CAF-1 p60	TMA CAF-1 p60 (%)	F-up
m	39	MM	≤/=1.00	IA	+++	37	+++	34	6 R
f	36	MM	≤/=1.00	IB	+++	33	++	29	4 N
m	56	MM	≤/=1.00	IB	++	24	++	23	2 NED
m	35	MM	≤/=1.00	IB	++	23	++	21	7 NED
m	41	MM	≤/=1.00	IB	++	25	++	23	7 NED
f	32	MM	≤/=1.00	IA	++	27	++	22	6 NED
f	66	MM	1.01–2.00	IB	+++	38	+++	35	12 N
f	47	MM	1.01–2.00	IIA	+++	55	+++	45	12 N,M,D
f	39	MM	1.01–2.00	IB	+++	46	+++	42	9 N
f	37	MM	1.01–2.00	IIA	+++	51	+++	47	7 R,N
f	43	MM	1.01–2.00	IB	++	23	++	21	9 NED
m	22	MM	1.01–2.00	IA	++	24	++	22	4 NED
m	37	MM	1.01–2.00	IA	++	26	++	20	3 NED
m	43	MM	1.01–2.00	IB	+++	40	+++	39	7 N
m	45	MM	2.01–4.00	IIIC	+++	49	+++	46	11 N,M,D
m	47	MM	2.01–4.00	IIB	+++	39	++	28	10 N
f	42	MM	2.01–4.00	IIA	+++	43	+++	37	9 N,M,D
f	46	MM	2.01–4.00	IIB	+++	40	+++	39	3 N
f	81	MM	2.01–4.00	IIA	+++	45	+++	41	2 M
m	32	MM	2.01–4.00	IIB	+++	47	+++	41	3 N,M
f	50	MM	2.01–4.00	IIA	++	25	++	22	11 NED
m	38	MM	2.01–4.00	IIA	++	25	++	24	3 NED
f	56	MM	2.01–4.00	IIA	++	24	++	23	13 NED
m	21	MM	2.01–4.00	IIB	++	27	++	22	14 NED
f	55	MM	>4.00	IIC	++	22	++	21	14 NED
m	54	MM	>4.00	IIB	++	21	++	20	12 NED
m	35	MM	>4.00	IIC	++	26	++	24	2 NED
f	38	MM	>4.00	IIC	+++	44	+++	38	1 N
f	52	MM	>4.00	IIC	+++	38	++	29	2 R,M,D
m	44	MM	>4.00	IIC	+++	49	+++	48	12 N

NED: not evidence of disease; R: relapse; M: distant metastasis; D: death for disease.

**Table 6:** Clinical and pathological features of the study population of LSCC, ordered by grading. Correlation with CAF-1 p60 expression.

Sex	Age	Histotype	Grading	TNM Stage	WS CAF-1 p60	WS CAF-1 p60 (%)	TMA CAF-1 p60	TMA CAF-1 p60 (%)	F-up
m	38	SCC	G1	II	++	22	++	21	187 NED
m	66	SCC	G1	IVA	+++	42	+++	33	184 M
m	53	SCC	G1	IVA	++	27	++	22	182 NED
m	68	SCC	G1	III	++	23	++	20	181 NED
m	70	SCC	G1	II	++	22	++	21	180 NED
m	70	SCC	G1	I	++	24	++	23	82 NED
m	53	SCC	G1	I	++	25	++	24	189 NED
m	76	SCC	G2	II	++	26	++	22	188 NED
m	65	SCC	G2	I	++	21	++	20	186 NED
m	75	SCC	G2	IVA	++	27	+++	31	182 NED
m	72	SCC	G2	IVA	++	23	++	21	58 NED
m	52	SCC	G2	I	+++	40	+++	38	45 M
m	61	SCC	G2	II	++	22	++	21	39 NED
m	65	SCC	G2	IVA	++	25	+++	31	38 NED
m	57	SCC	G3	IVA	++	26	++	25	69 NED
m	73	SCC	G3	III	++	25	++	24	66 NED
m	62	SCC	G3	III	++	23	++	22	45 NED
m	63	SCC	G3	IVA	++	23	++	22	186 NED
m	58	SCC	G3	IVA	++	25	++	24	185 NED
m	60	SCC	G3	I	++	24	++	23	153 NED
m	75	SCC	G3	II	++	22	++	21	65 NED
m	52	SCC	G3	IVB	+++	39	+++	37	187 M
m	66	SCC	G3	IVA	++	23	++	21	183 NED
m	59	SCC	G3	III	++	21	++	20	181 NED
m	75	SCC	G3	IVA	++	25	++	23	124 NED
m	58	SCC	G3	IVA	++	27	++	26	69 NED
m	71	SCC	G3	IVA	++	24	++	22	53 NED
m	50	SCC	G3	IVA	++	26	++	24	119 NED
m	72	SCC	G3	III	+++	38	+++	35	180 M
m	65	SCC	G3	IVA	++	28	++	27	124 NED

NED: not evidence of disease; R: relapse; M: distant metastasis; D: death for disease.

**Table 7:** Clinical and pathological features of the study population of SSCC, ordered by grading. Correlation with CAF-1 p60 expression.

Sex	Age	Histotype	Grading	TNM Stage	WS CAF-1/p60	WS CAF-1/p60 (%)	TMA CAF-1 p60	TMA CAF-1 p60 (%)	F-up
m	76	SCC	G1	I	++	23	++	21	223 NED
m	73	SCC	G1	I	++	24	++	23	222 NED
f	86	SCC	G1	I	++	22	++	20	221 NED
m	72	SCC	G1	II	++	26	++	22	218 NED
m	36	SCC	G1	II	++	24	++	23	209 NED
f	95	SCC	G1	II	++	22	++	21	122 NED
m	80	SCC	G1	II	++	26	++	23	69 NED
m	55	SCC	G1	I	++	24	++	23	69 NED
f	67	SCC	G2	II	++	22	++	22	210 NED
m	49	SCC	G2	II	+++	36	++	29	207 R,M
f	78	SCC	G2	IV	++	25	++	24	147 NED
f	83	SCC	G2	I	++	26	++	23	127 NED
m	80	SCC	G2	I	++	24	++	24	126 NED
f	68	SCC	G2	II	++	23	++	23	125 NED
m	79	SCC	G2	II	++	22	++	21	90 NED
f	51	SCC	G2	I	++	26	++	21	24 NED
m	63	SCC	G2	I	++	24	++	23	69 NED
m	59	SCC	G2	II	++	26	++	25	70 NED
m	85	SCC	G2	I	++	21	++	20	66 NED
m	32	SCC	G3	IV	++	22	++	21	213 NED
m	67	SCC	G3	II	+++	41	+++	40	212 R
f	71	SCC	G3	I	++	22	++	21	209 NED
f	76	SCC	G3	I	++	27	++	25	201 NED
f	72	SCC	G3	IV	++	23	++	22	189 NED
m	67	SCC	G3	II	++	24	++	23	155 NED
f	73	SCC	G3	II	++	22	++	21	12 NED
f	42	SCC	G3	III	++	21	++	20	12 NED
m	74	SCC	G3	II	++	27	++	26	68 NED
f	77	SCC	G3	II	++	24	++	24	64 NED
m	66	SCC	G3	II	++	25	++	24	216 NED

NED: not evidence of disease; R: relapse; M: distant metastasis; D: death for disease.

**Table 8:** Whole tissue sections and TMA concordance, *K*-coefficients grouped by pathology. Cohen’s weighted kappa statistic, standard error and 95% confidence intervals are shown.

	OSCC	PC	SM	SGT	LSCC	SSCC
***K*-coefficient**	0.8018	0.8148	0.8018	0.8529	0.8696	0.8076
**S.E.**	0.07361111	0.08611111	0.07361111	0.07638889	0.08819444	0.14513889
**95% CI**	0.593–1	0.571–1	0.593–1	0.627–1	0.620–1	0.374–1

## References

[1] Feinberg A.P., Ohlsson R., Henikoff S. (2006). The epigenetic progenitor origin of human cancer. Nat. Rev. Genet.

[2] Feinberg A.P., Tycko B. (2004). The history of cancer epigenetics. Nat. Rev. Cancer.

[3] Jones P.A., Baylin S.B. (2002). The fundamental role of epigenetic events in cancer. Nat. Rev. Genet.

[4] Jones P.A., Baylin S.B. (2007). The epigenomics of cancer. Cell.

[5] Ozanne S.E., Constancia M. (2007). Mechanisms of disease: The developmental origins of disease and the role of the epigenotype. Nat. Clin. Pract. Endocrinol. Metab.

[6] Schulz W.A., Hatina J. (2006). Epigenetics of prostate cancer: Beyond DNA methylation. J. Cell. Mol. Med.

[7] Li L.C. (2007). Epigenetics of prostate cancer. Front. Biosci.

[8] Liu S., Ren S., Howell P., Fodstad O., Riker A.I. (2008). Identification of novel epigenetically modified genes in human melanoma via promoter methylation gene profiling. Pigment Cell Melanoma Res.

[9] Egger G., Liang G., Aparicio A., Jones P.A. (2004). Epigenetics in human disease and prospects for epigenetic therapy. Nature.

[10] Heightman T.D. (2011). Therapeutic prospects for epigenetic modulation. Expert Opin. Ther. Targets.

[11] Sharma S.K., Wu Y., Steinbergs N., Crowley M.L., Hanson A.S., Casero R.A., Woster P.M. (2010). (Bis)urea and (bis)thiourea inhibitors of lysine-specific demethylase 1 as epigenetic modulators. J. Med. Chem..

[12] Rountree M.R., Bachman K.E., Herman J.G., Baylin S.B. (2001). DNA methylation, chromatin inheritance, and cancer. Oncogene.

[13] Sandoval J., Esteller M. (2012). Cancer epigenomics: Beyond genomics. Curr. Opin. Genet. Dev.

[14] Kouzarides T. (2007). Chromatin modifications and their function. Cell.

[15] Ehrenhofer-Murray A.E. (2004). Chromatin dynamics at DNA replication, transcription and repair. Eur. J. Biochem.

[16] De Koning L., Corpet A., Haber J.E., Almouzni G. (2007). Histone chaperones: An escort network regulating histone traffic. Nat. Struct. Mol. Biol.

[17] Tagami H., Ray-Gallet D., Almouzni G., Nakatani Y. (2004). Histone H3.1 and H3.3 complexes mediate nucleosome assembly pathways dependent or independent of DNA synthesis. Cell.

[18] Krude T., Keller C. (2001). Chromatin assembly during S phase: Contributions from histone deposition, DNA replication and the cell division cycle. Cell. Mol. Life Sci.

[19] Ramirez-Parra E., Gutierrez C. (2007). The many faces of chromatin assembly factor 1. Trends Plant Sci.

[20] Linger J.G., Tyler J.K. (2007). Chromatin disassembly and reassembly during DNA repair. Mutat. Res.

[21] Gaillard P.H., Martini E.M., Kaufman P.D., Stillman B., Moustacchi E., Almouzni G. (1996). Chromatin assembly coupled to DNA repair: A new role for chromatin assembly factor 1. Cell.

[22] Verger A., Crossley M. (2004). Chromatin modifiers in transcription and DNA repair. Cell. Mol. Life Sci.

[23] Staibano S., Mascolo M., Rocco A., Lo Muzio L., Ilardi G., Siano M., Pannone G., Vecchione M.L., Nugnes L., Califano L. (2011). The proliferation marker Chromatin Assembly Factor-1 is of clinical value in predicting the biological behaviour of salivary gland tumours. Oncol. Rep.

[24] Mascolo M., Vecchione M.L., Ilardi G., Scalvenzi M., Molea G., di Benedetto M., Nugnes L., Siano M., de Rosa G., Staibano S. (2010). Overexpression of Chromatin Assembly Factor-1/p60 helps to predict the prognosis of melanoma patients. BMC Cancer.

[25] Staibano S., Mascolo M., Mancini F.P., Kisslinger A., Salvatore G., di Benedetto M., Chieffi P., Altieri V., Prezioso D., Ilardi G. (2009). Overexpression of chromatin assembly factor-1 (CAF-1) p60 is predictive of adverse behaviour of prostatic cancer. Histopathology.

[26] Staibano S., Mignogna C., Lo Muzio L., Mascolo M., Salvatore G., di Benedetto M., Califano L., Rubini C., de Rosa G. (2007). Chromatin assembly factor-1 (CAF-1)-mediated regulation of cell proliferation and DNA repair: A link with the biological behaviour of squamous cell carcinoma of the tongue?. Histopathology.

[27] Polo S.E., Theocharis S.E., Klijanienko J., Savignoni A., Asselain B., Vielh P., Almouzni G. (2004). Chromatin assembly factor-1, a marker of clinical value to distinguish quiescent from proliferating cells. Cancer Res.

[28] Polo S.E., Theocharis S.E., Grandin L., Gambotti L., Antoni G., Savignoni A., Asselain B., Patsouris E., Almouzni G. (2010). Clinical significance and prognostic value of chromatin assembly factor-1 overexpression in human solid tumours. Histopathology.

[29] Chen Y., Miller C., Mosher R., Zhao X., Deeds J., Morrissey M., Bryant B., Yang D., Meyer R., Cronin F. (2003). Identification of cervical cancer markers by cDNA and tissue microarrays. Cancer Res.

[30] Fons G., Burger M.P., Ten Kate F.J., van der Velden J. (2007). Identification of potential prognostic markers for vulvar cancer using immunohistochemical staining of tissue microarrays. Int. J. Gynecol. Pathol.

[31] Gomaa W., Ke Y., Fujii H., Helliwell T. (2005). Tissue microarray of head and neck squamous carcinoma: Validation of the methodology for the study of cutaneous fatty acid-binding protein, vascular endothelial growth factor, involucrin and Ki-67. Virchows Archiv.

[32] Su Y. (2006). Immunohistochemical Expressions of Ki-67, Cyclin D1, -Catenin, Cyclooxygenase-2, and epidermal growth factor receptor in human colorectal adenoma: A validation study of tissue microarrays. Cancer Epidemiol. Biomark. Prev.

[33] Van den Eynden G.G., van der Auwera I., van Laere S., Colpaert C.G., van Dam P., Merajver S., Kleer C.G., Harris A.L., van Marck E.A., Dirix L.Y. (2004). Validation of a tissue microarray to study differential protein expression in inflammatory and non-inflammatory breast cancer. Breast Cancer Res. Treat.

[34] Wan W.H., Fortuna M.B., Furmanski P. (1987). A rapid and efficient method for testing immunohistochemical reactivity of monoclonal antibodies against multiple tissue samples simultaneously. J. Immunol. Methods.

[35] Kononen J., Bubendorf L., Kallioniemi A., Barlund M., Schraml P., Leighton S., Torhorst J., Mihatsch M.J., Sauter G., Kallioniemi O.P. (1998). Tissue microarrays for high-throughput molecular profiling of tumor specimens. Nat. Med.

[36] Monteiro L.S., Diniz-Freitas M., Garcia-Caballero T., Forteza J., Fraga M. (2010). EGFR and Ki-67 expression in oral squamous cell carcinoma using tissue microarray technology. J. Oral Pathol. Med.

[37] Boone J., van Hillegersberg R., van Diest P.J., Offerhaus G.J., Rinkes I.H., Kate F.J. (2008). Validation of tissue microarray technology in squamous cell carcinoma of the esophagus. Virchows Arch.

[38] Hecht J.L., Kotsopoulos J., Gates M.A., Hankinson S.E., Tworoger S.S. (2008). Validation of tissue microarray technology in ovarian cancer: Results from the Nurses’ Health Study. Cancer Epidemiol. Biomark. Prev.

[39] Alkushi A. (2009). Validation of tissue microarray biomarker expression of breast carcinomas in Saudi women. Hematol. Oncol. Stem Cell Ther.

[40] Cunha K.S., Caruso A.C., Goncalves A.S., Bernardo V.G., Pires A.R., da Fonseca E.C., de Faria P.A., da Silva L.E., Geller M., de Moura-Neto R.S. (2009). Validation of tissue microarray technology in malignant peripheral nerve sheath tumours. J. Clin. Pathol.

[41] Camp R.L., Neumeister V., Rimm D.L. (2008). A decade of tissue microarrays: Progress in the discovery and validation of cancer biomarkers. J. Clin. Oncol.

[42] Sullivan C.A., Chung G.G. (2008). Biomarker validation: *In situ* analysis of protein expression using semiquantitative immunohistochemistry-based techniques. Clin. Colorectal. Cancer.

[43] Giltnane J.M., Rimm D.L. (2004). Technology insight: Identification of biomarkers with tissue microarray technology. Nat. Clin. Pract. Oncol.

[44] Wang S.L., Yang C.H., Chen H.H., Chai C.Y. (2006). A simple and economical method for the manual construction of well-aligned tissue arrays. Pathol. Res. Pract.

[45] Eguiluz C., Viguera E., Millan L., Perez J. (2006). Multitissue array review: A chronological description of tissue array techniques, applications and procedures. Pathol. Res. Pract.

[46] Edge S.B., Byrd D.R., Compton C.C., Fritz A.G., Greene F.L., Trotti A (2010). AJCC Cancer Staging Manual.

[47] Hoos A., Urist M.J., Stojadinovic A., Mastorides S., Dudas M.E., Leung D.H., Kuo D., Brennan M.F., Lewis J.J., Cordon-Cardo C. (2001). Validation of tissue microarrays for immunohistochemical profiling of cancer specimens using the example of human fibroblastic tumors. Am. J. Pathol.

[48] Kundel H.L., Polansky M. (2003). Measurement of observer agreement. Radiology.

